# Recent Developments in Home Sleep-Monitoring Devices

**DOI:** 10.5402/2012/768794

**Published:** 2012-10-14

**Authors:** Jessica M. Kelly, Robert E. Strecker, Matt T. Bianchi

**Affiliations:** ^1^Department of Neurology, Massachusetts General Hospital, 55 Fruit Street, Wang 720, Boston, MA 02114, USA; ^2^VA Boston Healthcare System and Harvard Medical School, Brockton, MA 02301, USA

## Abstract

Improving our understanding of sleep physiology and pathophysiology is an important goal for both medical and general wellness reasons. Although the gold standard for assessing sleep remains the laboratory polysomnogram, there is an increasing interest in portable monitoring devices that provide the opportunity for assessing sleep in real-world environments such as the home. Portable devices allow repeated measurements, evaluation of temporal patterns, and self-experimentation. We review recent developments in devices designed to monitor sleep-wake activity, as well as monitors designed for other purposes that could in principle be applied in the field of sleep (such as cardiac or respiratory sensing). As the body of supporting validation data grows, these devices hold promise for a variety of health and wellness goals. From a clinical and research standpoint, the capacity to obtain longitudinal sleep-wake data may improve disease phenotyping, individualized treatment decisions, and individualized health optimization. From a wellness standpoint, commercially available devices may allow individuals to track their own sleep with the goal of finding patterns and correlations with modifiable behaviors such as exercise, diet, and sleep aids.

## 1. Introduction

 The laboratory polysomnogram (PSG) has long been the gold standard for assessing sleep physiology in health and disease. The PSG has proven most useful for the diagnosis and treatment of obstructive sleep apnea (OSA), although less common disorders are also readily identified by laboratory PSG, including narcolepsy, rapid-eye-movement (REM) sleep behavior disorder, non-REM parasomnias, and periodic limb movements of sleep. The diagnostic criteria for certain sleep disorders, such as restless legs syndrome and insomnia, are purely clinical. For insomnia in particular, exclusive reliance on self-reported complaints presents two important challenges in understanding this common disorder. First, it is not uncommon to observe a mismatch between the subjective report of sleep-wake durations and the objective findings of the PSG. Second, the lack of objective data constrains the capacity to phenotype insomnia patients, a limitation that has implications ranging from epidemiology studies to the development of therapeutic strategies.

 Despite the clear utility of PSG in clinical sleep medicine, issues of cost and inconvenience have motivated the development of portable devices capable of evaluating sleep in the home. Clinical home testing is currently targeting sleep disordered breathing, and the data supporting the use of home sleep apnea devices has been reviewed recently [1, 2]. Although, to date, home-based sleep measurements have focused on sleep apnea diagnostics, patients with insomnia may also benefit from advances in home-sleep-monitoring devices. Wrist actigraphy, which measures limb movement, has been used for several decades in various contexts, including in patients with insomnia; however, actigraphy is not widely used clinically. For example, recent practice parameters suggest actigraphy as an option for circadian rhythm disorders and potentially for adjunctive assessment of insomnia [3]. However, the diagnostic classifications of insomnia provided by the American Academy of Sleep Medicine and by the psychiatric Diagnostic and Statistical Manual do not include any objective criteria. Despite the extensive published experience with wrist movement-based monitors in research settings [4, 5], actigraphy does not enjoy wide clinical use because of limited utility outside of circadian rhythm disorders, which are often evident by clinical history alone. Nevertheless, commercially available movement-based sleep monitors are growing increasingly common in the consumer wellness market. The utility of such devices in a medically unregulated fashion remains uncertain. 

 The wellness and clinical markets share an interest for improved metrics of “real-world” sleep patterns. Longitudinal home monitoring avoids certain limitations of the laboratory PSG, such as the atypical sleeping environment and the single-night snapshot. Sleep is a dynamic process that varies from day to day, and hence it is important to measure multiple nights of sleep for medical, research, and wellness reasons. Home monitoring devices offer the potential to provide a more realistic platform in which many nights of sleep data can be captured. Longitudinal data is likely to prove invaluable for the discovery of intrinsic patterns of sleep variability or to correlate sleep with the timing of various other activities such as exercise, naps, food, caffeine, alcohol, and stress. Since each of these “other activities” can vary from day to day, complex effects and interactions are expected to occur, which creates the need for large data sets to identify correlations with sleep. Quantifying sleep in relation to these diverse factors can only be accomplished through longitudinal data, with the clinical goal of individualized evaluations and treatment strategies. The personal wellness goal of sleep-monitoring in order to optimize health also stands to be achieved through longitudinal monitoring and self-tracking. This goal is served by portable monitoring in a variety of contexts outside of the field of sleep medicine (for review, see [6]).

 The present paper reviews recent developments in the area of devices that can be used for home-based sleep assessment, some of which are currently available for direct purchase in the wellness market. Devices developed explicitly for sleep quality monitoring are reviewed, as are devices developed for other reasons that could potentially be adapted for sleep-monitoring. The list is not intended to be exhaustive, and it is likely that the field will continue to expand rapidly as new devices are introduced. We do not review devices used for detection or diagnosis of sleep apnea [1], nor do we review standard actigraphy devices [7]. When available, validation information is provided (see Section 7 for further comments on the metrics of sensitivity, specificity, and accuracy). These devices are grouped into categories based on the type of data collected. For each device, listed alphabetically within category, the key features are evaluated including availability of published validation studies. Finally, we discuss a research agenda for the field of home-based sleep-monitoring. This paper is not intended to endorse any particular device or to advise readers medically regarding diagnostics or therapeutics; in fact it is important to recognize that nonrefreshing sleep can be associated with numerous medical and psychiatric conditions, and physician consultation is suggested for concerned readers. 

## 2. Sleep-Monitoring Based on Brain Activity Signals

### 2.1. iBrain (NeuroVigil)

 This device consists of headgear that records a single frontal lead EEG. The algorithm used to process the frontal EEG is based originally on work done in zebra finch birds, in which 84% accuracy was obtained when compared to manual sleep-wake scoring in that species [8]. The company web site indicates ongoing human studies; however, no validation studies in humans are currently available. Data from the device can be updated via a USB drive which also charges the device. The device can record multiple nights of data. 

### 2.2. *Zeo* (Available for Consumer Purchase)

 This device consists of an elastic headband with fabric sensors on the forehead that detect a combination of electroencephalogram (EEG), frontalis muscle electromyogram (EMG), and electrooculogram (EOG) signals. The headband broadcasts wirelessly to either an alarm clock receiver station or to an iPhone for analysis. The main advantage of the Zeo is the capacity to monitor sleep over time with relative ease. Indeed, the headband sensor can be used daily for several months before the sensor pads require replacement. 

 A proprietary neural network model uses the data streams to render classifications of wake, light NREM, deep NREM, and REM sleep in 30-second epochs. Deep NREM sleep corresponds to slow wave sleep or stage N3. The term “deep” is often linked to this stage because of the conspicuous high-amplitude and low-frequency EEG signal pattern and because awakening from this stage of sleep is most difficult. Although the Zeo algorithm assigns greater weight in their sleep quality index to this stage of sleep, there is little evidence in the literature that the amount of “deep” sleep correlates with feeling refreshed. Deep sleep has been shown to correlate with “homeostatic” sleep pressure: the longer one has been awake, the more sleep pressure accumulates and the more deep sleep is observed during subsequent recovery sleep.

 The light NREM sleep class is actually a combination of two stages of NREM sleep known as N1 and N2. However, these are fundamentally different states, with the latter containing two classic features of sleep, known as sleep spindles and K-complexes, while the former lacks these features and is instead characterized by mild slowing of the EEG (relative to high-frequency and low-amplitude EEG signals typical of wakefulness). In fact, stage N2 represents the majority of sleep time in a normal individual. It is worth emphasizing that there is no clinical or biological basis for combining these two stages, and the potentially negative connotation of the term “light” may give users the false impression that sleep scored in this category is necessarily abnormal. Although excessive stage N1 sleep may indicate sleep fragmentation (of any cause), this is not the case for stage N2. There is no way to distinguish N1 and N2 using this device, due to combining N1 and N2 sleep into a single category. 

 Zeo recently published a validation study of their sleep staging algorithm, which was initially optimized using a group of healthy adults aged 21–60 (67% male) and then tested in a separate group of 26 healthy adults aged 19–60 years (50% male) [9]. Subjects underwent laboratory PSG with simultaneous headband monitoring, which showed ~75% agreement on epoch-by-epoch scoring across all sleep-wake stages. Of note, the two human experts scoring the PSG data showed only 83% agreement, which can be viewed as an apparent upper limit of performance for any automated scoring algorithm. This modest agreement is similar to prior literature and serves as a reminder that sleep stage scoring is characterized by substantial uncertainty. When considering each PSG-scored sleep-wake stage individually, Zeo correctly identified 71% of deep NREM sleep and 64% of wake, while it was better at detecting light NREM and REM sleep (86% each). Another way to understand the accuracy of the classification is to ask how likely a stage reported by the Zeo matches that defined by the PSG scoring. For example, there was a 75% chance that an epoch scored as REM by the Zeo was correct. When Zeo was mistaken about REM sleep, the most likely PSG-defined stages to be misclassified as REM were light NREM and wake. Epochs scored as deep NREM had a 69% chance of being correct, while those scored as wake or REM sleep each had ~85% chance of being correct. 

The extent to which the classifier algorithm retains accuracy with patients suffering from medical, neurological, psychiatric, or sleep disorders is unknown. One might expect certain medications to alter accuracy, due to effects on the EEG, EMG, and EOG (especially neuroactive medications). In addition, the effects of caffeine, smoking, and alcohol (all of which are known to affect sleep physiology) also remain unknown. Zeo does have the option for researchers to pursue off-line postprocessing of the recorded signals. 

## 3. Sleep-monitoring Based on Autonomic Signals

### 3.1. Heally Recording System

The Heally system consists of a shirt with a combination of embedded sensors and wired adhesive electrodes that measure respiratory and cardiac physiology, as well as ports for optional EMG and EOG electrodes [10]. A small study of six healthy male subjects was conducted at home over multiple nights, in which sleep versus wake was scored according to nonvalidated criteria (a human scorer classified sleep-wake state using a combination of video, EOG and EMG signals). Like wrist actigraphy, the shirt overestimated total sleep time as well as the number of brief awakenings, compared to the human scoring. The accuracy across subjects was modest at approximately 80% agreement with human scoring, similar to accuracies obtained with limb actigraphy [10].

### 3.2. M1 (SleepImage)

 This medical device consists of a small processing unit and wire electrode that attaches to the chest via adhesive pads. Data signals stored locally in the device include electrocardiogram (ECG), actigraphy, and body position. The trunk actigraphy signal is used to determine total sleep time, sleep efficiency, and the number of awakenings that occur within sleep. The signals are subjected to off-line analysis through the SleepImage web site. The ECG component is used to compute cardiopulmonary coupling frequencies, a metric that consists of a combination of respiratory-driven heart rate variability (autonomic function) and fluctuations in the R-wave amplitude that relate to mechanical changes of breathing (position of the heart and lung tissue relative to the skin surface) [11]. This algorithm distinguishes “stable” versus “unstable” NREM sleep, using the cardiopulmonary coupling metric rather than the brain, eye, and muscle activity used for the standard classification of N1, N2, N3, and REM sleep. The relationship between “stable” and “unstable” NREM sleep and conventional EEG-derived sleep stages is described next. 

 Stable NREM is associated mainly with stage N3 but also includes portions of N2 and is associated with a coupling frequency in the range of the normal respiratory rate, which is around 0.3 Hz. This pattern is known as high-frequency coupling (HFC). Unstable NREM sleep is associated mainly with stage N1 but also portions of stage N2, especially when N2 sleep is fragmented and/or the “cyclic alternating pattern” is seen [12]. This pattern is associated with coupling in a lower range (0.1 Hz) and is known as low-frequency coupling (LFC). REM sleep and wakefulness produce similar coupling frequencies, due to similarly irregular breathing. This pattern is known as very low-frequency coupling (VLFC) and occurs at frequencies under 0.01 Hz. 

When sleep apnea is present, the contribution of the LFC component is increased, known as elevated LFC or e-LFC. Within this e-LFC metric, if the frequency is variable, this is known as broad-band coupling and is associated with obstructive sleep apnea. This pattern corresponds to the observation that obstructive apnea events typically have variable cycle lengths. However, when the dominant e-LFC values are very similar over time, this is known as “narrow band coupling” and is associated with central sleep apnea which typically has a short and “metronomic” cycle length. Thus, although the device is not approved for the diagnosis of sleep apnea, within known sleep apnea patients some distinction can be achieved between obstructive and central phenotypes [13]. It is worth noting that sleep that is highly fragmented for a variety of reasons may be dominated by a high percentage of the night spent in the LFC pattern. 

 The M1 can be used for 5–7 nights of recording on two disposable button batteries. The raw ECG data is stored locally in the device and is extracted off-line for analysis. Early validation studies took advantage of the fact that the coupling algorithm can be applied to any ECG signal, such as those obtained routinely in overnight PSG studies. Analysis of the large Sleep Heart Health Study database showed correlations of HFC and LFC with important factors such as stroke and hypertension [14]. Subsequent studies showed correlations of coupling metrics with depression and fibromyalgia [15, 16].

 One limitation of the device is that certain patient populations may not be amenable to ECG analysis, including those with certain types of arrhythmias and potentially patients with autonomic dysfunction. Also, trunk actigraphy such as that provided by this device does not have as much supporting data, compared to the traditional wrist actigraphy, for estimating sleep and wake. 

## 4. Sleep-monitoring Based on Movement

### 4.1. *Fitbit *(Available for Consumer Purchase)

The Fitbit monitor is a small device that can be worn on the wrist, clipped to clothing, or carried in a pocket. The features include a pedometer and altimeter (to count steps or hills climbed), a calorie counting feature (extrapolated from the estimate of steps walked), movement detection by actigraphy, and a clock. The analysis of movement yields standard sleep-related metrics such as a distinction between sleep and wake, total sleep time, sleep latency, and an “arousal index” based on episodes of movement during presumed sleep time. There are no published validations of the accuracy of the sleep-wake metrics of the *Fitbit* compared to PSG or to standard actigraphy watch devices. 

### 4.2. *Lark *(Available for Consumer Purchase)

 The Lark device is a wrist-watch actigraphy monitor that features a silent vibrating alarm. Actigraphy metrics include total sleep duration, sleep latency, and a “sleep quality index” based on movements. However, there are no published validation reports comparing *Lark*-derived measures with standard wrist actigraphy or PSG data. The device currently requires an iPhone or iPad or iTouch to visualize the data, although the web site indicates that an Android platform is under development. 

### 4.3. *Sleep Cycle Alarm *(Available for Consumer Purchase)

The Sleep Cycle alarm clock is an iPhone application that uses the built-in accelerometer of the iPhone to monitor movement during the night. The iPhone is placed near one's pillow. The application reports graphs of total sleep time and a distinction between light sleep, deep sleep, and wake. There are no available studies of the device to validate this analysis of sleep. The application also has a smart-alarm feature to wake users within thirty minutes of their final alarm by detecting periods of light sleep based on movement. Like the other devices making this smart-alarm claim, supporting validation studies are not available. 

### 4.4. SleepTracker (Innovative Sleep Solutions) (Available for Consumer Purchase)

 The SleepTracker device is a wrist watch that records movement based on actigraphy. Like similar movement-based devices, the web site claims a smart-alarm feature that determines optimal points within sleep to awaken to feel refreshed. The watch has audio-alarm and vibrating-alarm options. Sleep data can be viewed through the web site following USB upload, including total sleep time and a metric of “sleep quality” based on movement. Although there are no published validation studies of the smart-alarm feature or the sleep-wake accuracy, the company has performed testing in 18 adults who underwent simultaneous sleep laboratory monitoring for suspected sleep apnea (unpublished data, personal communication with Lee Loree, owner). In this study, the device was >90% accurate in detecting events of sleep disruption, but the relationship of the detected events to clinically defined sleep parameters is untested. 

### 4.5. *Up (Jawbone) *(Available for Consumer Purchase)

 The Up monitor by Jawbone is a bracelet-like device that interacts with the iPhone. The device serves as a pedometer, and although it reports a distinction between “deep" and “light" sleep, there are no published validation studies that compare the device to PSG or to wrist actigraphy, and even standard actigraphy algorithms do not typically allow such a distinction. The device also includes a smart-alarm feature that claims to awaken the wearer at the "optimal" time, but, again, this commonly reported feature lacks published validation. 

### 4.6. *WakeMate *(Available for Consumer Purchase)

WakeMate is a wristband device that transmits actigraphy data to a smart phone to report basic sleep metrics such as total sleep time, sleep latency, number of awakenings, and a “sleep quality” score based on movements. Compatible interfaces include iPhone, Android, and Blackberry phones. Like the above devices, it also makes the smart-alarm claim to determine the optimal wake time within a window ending in the final alarm setting. The website indicates that the device is 95%–98% as accurate as standard actigraphy. However, supporting validation data is not available for either of these claims. 

## 5. Bed-Based Sleep Monitors

### 5.1. Air Cushion

This is a thin, air-filled cushion designed to be positioned on top of a mattress [17]. The pressure-sensing pad records heart rate, respiration rate, snoring, and body movement. An automated sleep staging algorithm using heart rate and movement signals was developed based on 27 overnight recordings from eight university students who had no subjective sleep complaints. The algorithm demonstrated the following agreement with PSG data: 82.6% for NREM sleep, 38.3% for REM sleep, and 70.5% for wake. As is commonly the case with autonomic metrics, REM sleep and wake were difficult to distinguish. 

### 5.2. EarlySense Mattress

This device is a piezoelectric sensor that is placed under a mattress. The system measures respiration, heart rate, snoring, coughing, and movement. In a study available on their website of 40 children and 16 adults (who were being evaluated for sleep complaints), a Bayesian classifier algorithm that combined features of respiration with movement signals distinguished sleep versus wake with modest accuracy compared to concurrent PSG scoring. On an epoch by epoch basis, sleep was detected with a sensitivity of 84% but a specificity of only 30% (compared to wake); wake was more accurately identified (sensitivity of 68% and specificity of 80%). Further distinction of REM versus NREM sleep was also described, but statistics of accuracy are not presented. However, REM was reportedly misassigned to periods of light NREM sleep and adjacent wake epochs. 

### 5.3. Emfit Bed Sensor

This system consists of Emfit foil electrodes placed underneath a foam mattress which record movement, respiratory rate, and heart rate data [18–20]. These data streams were then subject to machine learning algorithms to optimize agreement with human PSG scoring in a sample of 17 healthy adults. The mattress algorithm showed an agreement of 71% with PSG data on an epoch by epoch basis [19]. Wake and REM sleep were most challenging to distinguish, as these two states were most often misclassified. In a similar study using the Emfit foil electrodes, sleep staging of eleven healthy female participants was moderately accurate compared to PSG, with an agreement of 76% [18]. In a separate study of nine females, the Emfit bed sensor was found to have a 79% agreement with PSG data in determining wake, NREM, and REM sleep states, but REM sleep was again difficult to classify [20]. 

### 5.4. Home Health Station (TERVA)

 The Home Health Station is a comprehensive system intended to be set up in a patient's home to record and display blood pressure, axillary temperature, respiration rate, heart rate, activity, and subjective behavioral diary entries [21]. The system includes a static-charge-sensitive bed engineered by Biomatt Monitoring systems, which measures heart rate, respiration rate, and time spent in “quiet” sleep based on movement data. Previous studies have found the accuracy of the static-charge-sensitive bed to be between 86% to 98% for classifying wake versus sleep [22]. In addition, the bed sensor has been used to detect sleep apnea: it detected sleep-disordered breathing during 4% of the night in healthy patients compared to 43% of the night in patients with known sleep apnea [23, 24].

### 5.5. Linen Sensor

 This system consists of electrodes embedded in the pillow case as well as the linens near the foot of the bed [25]. Validation was conducted in 30 patients undergoing sleep evaluation for a variety of clinical reasons, as well as six healthy subjects. Data quality was a concern in their study, as 20% of the recording time was not usable due to excess movement and/or poor contact with the sensors. In the six healthy subjects, the bed sensor classified 82% of the night as NREM sleep and 19% of the night as REM sleep, and this was relatively accurate compared to standard PSG, which classified 78% and 23% of night as NREM and REM sleep, respectively. The number of arousals (which were not defined in the paper) was underestimated compared to standard PSG data.

### 5.6. SleepMinder (BiancaMed)

This device is a radiofrequency monitor that uses 5.8 GHz frequencies to detect body movements [26]. The SleepMinder was studied by placing the sensor above and lateral to the bed, such as on a bedside table. It was most accurate when placed within 0.5 meters of the bed, with a maximum distance of 2.5 meters. Distinguishing sleep and wake showed 78% accuracy in a population of 153 subjects who underwent PSG monitoring for suspected sleep apnea. Total sleep time was overestimated, which is commonly the case with movement detection by wearable actigraphy devices. The device performance was less accurate in distinguishing wake, REM, and stage N1 sleep but reported 96% accuracy in classifying slow wave sleep. In a separate study of 176 patients who underwent overnight PSG monitoring for suspected sleep apnea, the device was able to classify subjects with versus without sleep apnea, based on a cutoff value of AHI =15, with a sensitivity of 89% and a specificity of 92% [27].

### 5.7. Touch-Free Life Care (TLC) System

The TLC system is a bed sensor that can transmit information for remote monitoring. This device can be placed underneath any standard mattress and wirelessly transmits heart rate, respiratory rate, and movement data. A sleep “quality score” is generated based on a combination of sleep duration, restlessness, heart rate, and breathing rate. However, validation studies of this sleep quality metric are not available. 

## 6. Other Devices with Potential for Sleep-monitoring 

### 6.1. BioHarness (Zephyr)

 This vest-like device is strapped across the chest and records respiration rate, heart rate, skin temperature, motor activity, and body position. The data can be wirelessly transferred for remote monitoring. 

### 6.2. HealthVest (SmartLifeTech)

 This is a one piece garment with electrodes embedded in the shirt. Respiration rate, heart rate, and body position can be measured and monitored remotely. 

### 6.3. LifeBed (Hoana)

 This is a bed used in clinical settings that displays and records respiration and heart rate, and it also alerts caretakers when a patient is out of bed. 

### 6.4. LifeShirt (VivoMetrics, Rae Systems)

This monitor is a form-fitting garment that measures multiple aspects of respiratory and cardiac physiology, movement, skin temperature, and body position, through a combination of sensors embedded in the fabric in combination with either dry [28] or adhesive electrodes [29], including the capacity for remote monitoring by wireless transmission. There are optional ports for extending monitoring to other specialized signals such as oximetry and blood pressure. Although the authors describe preliminary findings regarding the use of the shirt for sleep staging, no validation data is currently available. 

### 6.5. Magic Vest (Foundation Don Gnocchi)

 The vest is a form-fitting combination of cotton and lyrica to facilitate a close connection between the skin and nonadhesive ECG electrodes. The ECG signal tracking was reliable for detecting normal and abnormal rhythms compared with standard ECG [30]. The removable transmitter attaches to the vest and is about the size of a cell phone.

### 6.6. Radiofrequency Monitor

Radiofrequency monitors have been developed to detect movements of the sleeping subject as a means for unobtrusive monitoring. Li et al. studied the use of microwave band signals (26.5–40 GHz) [31], which are often used in radar detection. An antenna underneath the bed transmits radiofrequencies to a nearby receiver (i.e., a laptop) and allows long-term, overnight respiration and heart rate monitoring. The accuracy of the device in measuring heart rate was about 80% compared to finger tip pulse sensor, depending on body position. Of note, the accuracy of heart rate determination was compromised by nonsupine body position. The system is proposed as a way to monitor patients at home for sleep apnea, but such algorithms have yet to be developed. 

### 6.7. SenseWear Armband (BodyMedia)

 This device uses a combination of sensors including an accelerometer as well as sensors for heat flux, temperature, and galvanic skin response. Heart rate variability, body temperature, and other recoded measurements are used to determine wake, sleep onset, and total sleep time. In a small study on self-identified normal sleepers available on their web site, the armband agreed 85.3% of the time with PSG data in determining sleep versus wake states. 

### 6.8. SmartShirt (Sensatex)

 The SmartShirt is a cotton tee-shirt which uses sensors inside the fabric to measure and transmit real-time data of heart rate, body temperature, and movement. 

### 6.9. Shirt Monitor (Universidad Carlos III de Madrid)

 This smart shirt contains embedded electrodes to measure cardiac and pulmonary physiology and also includes a global-positioning system. Body temperature and position as well as geographic location can be monitored, the latter feature having high enough spatial resolution for application to hospital patients. An extended version of the device uses imbedded electrodes to monitor and wirelessly transmit vital signs as well as body position and temperature. The shirt is machine washable. 

### 6.10. Smart Shirt (Numetrex)

This line of clothing contains sensing fibers knitted into the fabric. Sensors record heart rate for viewing on the accompanying watch receiver.

### 6.11. V-Patch and Aingeal Devices (Intelesens)

 Intelesens develops wearable vital sign monitors for home and hospital use. The V-patch is an adhesive device that records 3-lead EKG for up to 7 days. The Aingeal is an adhesive device that records cardiac, respiratory, actigraphy, and temperature metrics for up to 48 hours of monitoring in hospitalized patients via a nearby bay station. 

### 6.12. Wealthy (http://www.wealthy-ist.com/)

 This device is a tight-fitting garment which uses impedance pneumography to determine respiration, piezoresistive sensors and accelerometers to determine movement and position, as well as sensors to track body temperature and heart rate. The shirt can store data locally or transmit data via bluetooth. 

### 6.13. WristCare (Vivago)

This wrist device has a four-day subject-specific activity adaptation period after which it tracks movement, skin temperature, and skin conductivity as well as the location of a patient in the hospital and remotely transmits the data. The device was designed as an automatic alarm device for the elderly and chronically ill. In a study of 28 adults, WristCare overestimated total sleep time by 59 minutes (whereas wrist actigraphy only overestimated total sleep time by 41 minutes) in comparison to PSG data [32].

### 6.14. Wrist Device (AMON)

 This device is a wristband which remotely transmits heart rate, blood pressure, oxygen saturation, and skin temperature. In addition, it has an accelerometer and can function as an automatic alarm [33]. The device was tested in 33 healthy adult volunteers during wakefulness; compared to standard laboratory devices, it had varying degrees of accuracy when measuring blood pressure, oxygen saturation, and heart rate [33]. 

### 6.15. Zio (http://www.irhythmtech.com/)

 The Zio consists of a small patch with two electrodes worn on the chest that enables cardiac monitoring for up to fourteen days. The disposable patch is intended for clinical use in patients with cardiac complaints, similar to a Holter monitor. Zio is designed to record cardiac arrhythmias and heart rate.

## 7. Discussion

Wearable monitors and passive off-body sensors are growing in popularity as novel strategies for recording and transmitting various physiological signals [6, 34, 35]. Their medical and wellness applications are vast and include the measurement of sleep patterns and potentially sleep quality. However, research must parallel this expanding arena to ensure appropriate validation studies and understanding of each device's limitations in order to maximize the utility of home monitoring. Validation is a costly and time-consuming process, and we discuss here various considerations for the design and testing of home sleep monitors, in hopes of providing a research agenda going forward. Peer reviewed studies remain the gold standard in the biomedical community, yet the claims of health and wellness devices have not been universally held to similar standards. This is crucial in the field of sleep monitors, since disturbed or nonrefreshing sleep may be associated with a number of medical and psychiatric disorders and may warrant physician consultation. We propose topics here to consider as a framework for a research agenda in this expanding field. 

### 7.1. Hardware Considerations in Sleep-monitoring

 The two major considerations are cost and comfort, as these may be the main factors limiting the scope of implementation. From a cost perspective, both up-front costs and ongoing costs (disposable parts or software/web site access) should be considered. The part of the body involved in contact-based devices (headband, wristband, shirt, etc.) may influence factors such as comfort and the integrity of recording. Whether the device can fall off or be influenced by subject placement should be considered—importantly, these factors may differ from person to person. Battery life and method/frequency of recharging may also play a role in consumer acceptance, especially for devices designed for long-term repeated monitoring. Finally, it is critical to consider the resilience to various factors present in the sleep environment, such as body movements, sweating, temperature, humidity, and the bed partner (e.g., the bed partner's movements, sounds, or sleep disorders may influence the data collected). 

### 7.2. Software Considerations in Sleep-monitoring

 The main consideration in sleep-monitoring software is ease of use. The manner of data access may influence user acceptance. In some cases, the data is processed for output analytics but not stored in its primary form. This has advantages of minimizing data storage needs and may be optimal for real-time analysis and use in the field. Although storing the raw data for off-line analysis has the advantage of facilitating algorithm improvements, the need for storage space and/or frequent uploading to a server may be cumbersome. Given the wide variety of sleep problems and comorbidities, it may be difficult if not impossible to have a single algorithm that is applicable across diverse populations, and thus it seems highly useful to have the raw data available for ongoing improvements. 

### 7.3. Utility of Objective, Longitudinal Monitoring

 The clinical paradigm of monitoring in sleep patients involves two main strategies: the laboratory PSG and the home diary. The PSG is information rich but has serious limitations of the unnatural environment and the single-night snapshot. The diary approach captures a person's experience in their home environment in a longitudinal manner but lacks objectivity. Home sleep monitors offer the potential to bridge these two extremes by providing some objective measures over time, ideally in parallel with subjective diary reports. In this way, patterns may be revealed in sleep and symptoms at the individual level. The longitudinal aspect allows analysis over multiple time scales, as certain people may have fluctuations in their sleep or symptoms over days, weeks, months, seasons, menstrual cycles, and so forth. To the extent that variability can be found, it then becomes possible to link this variability to behaviors such as caffeine, alcohol, medications, stress, and exercise—in principle any factor the individual may care to measure in hopes of finding sleep correlations. If correlations can be identified, this opens the opportunity to implement personalized behavioral modification plans to optimize sleep. For some individuals, certain medical or psychiatric treatment may be undertaken to improve sleep, and a home monitor may provide an adjunctive outcome measure in parallel with subjective response. There is even data to suggest that simply providing feedback to individuals with sleep problems, through objective sleep measurements, can improve subjective sleep complaints [36]. Finally, from a research and progress standpoint, having the capacity to add objective sleep measurements holds promise for improving the ability to phenotype sleep disorders such as insomnia that currently have purely subjective criteria. Such improvements could theoretically contribute to improved understanding of which types of treatments (prescriptions or alternative therapies or behavioral interventions) may be most beneficial. 

### 7.4. A Comment on Device Validation

 Validating a home sleep monitor involves comparing the performance to some other measurement. When this comparison involves the gold standard laboratory PSG, which is scored manually by experienced technicians, it is important to recognize that this gold standard is itself imprecise. Depending on the study, the interscorer reliability may be approximately 85%. This sets an upper limit on what can be expected of an automated algorithm (e.g., see the validation study of the Zeo device, in which two human scorers were used [9]). Furthermore, scoring reliability, and by extension, automated device scoring, may be influenced by the presence of sleep disorders such as sleep apnea or of factors that influence the aspect of sleep physiology measured by a home monitor. For example, an actigraphy device would need to be separately validated in patients with versus without intrinsic movement disorders (such as Parkinson's disease). Ideally, validations should include a spectrum of subject characteristics (age, sex, BMI, and health status), to improve the generality of use.

 The American Academy of Sleep Medicine scoring criteria utilizes a time interval of 30 seconds to define an epoch of sleep, with a “majority rules” approach to assigning a stage to an epoch that contains features of more than one stage [37]. Thus, if a device utilizes a time interval that is either shorter or longer than the AASM criteria, the validation results may differ. For example, shorter epochs may capture nuances of sleep architecture, while longer epochs or smoothing algorithms may yield a different image of sleep physiology. 

 It is worth mentioning that the term “accuracy” may carry several meanings. The standard manner of reporting diagnostic tests in medicine involves the sensitivity and specificity when tested against a gold standard. In diagnostic tests, typically one considers a disease to be either present or absent and a test result to be either positive or negative. In that setting, sensitivity refers to the portion of patients with the disease who test positive and specificity refers to the portion of patients without the disease who test negative. Accuracy is a term that incorporates sensitivity and specificity but is strongly dependent on the actual number of disease versus healthy individuals being tested (i.e., the prevalence or prior probability of disease). Specifically, accuracy refers to the sum of true positives and true negatives divided by all tested subjects. In the measurement of sleep, one can consider the analysis framework as follows: instead of disease presence versus absence, the diagnostic device indicates the presence or absence of sleep. For example, the portion of true sleep epochs (defined, e.g., by PSG) that are correctly classified by a device as sleep can be called the device sensitivity, while the portion of true wake epochs correctly classified by a device as wake can be called the device specificity. If the recording time were split evenly between wake and sleep (50% each), one could interpret the accuracy because the evenly divided time avoids a prevalence bias in the expected number of true positives and true negatives. However, for most individuals, wake is such a small part of time in bed that the composite accuracy metric may be dominated by the sensitivity of the device, especially if sensitivity and specificity values are dissimilar. Put another way, if test subjects sleep >95% of time in bed, a device can report high accuracy if it can correctly identify sleep epochs most of the time even if it labels most wake periods as sleep (but the opposite is not true). 

### 7.5. A Comment on “Normal Sleep”

 Although it is commonly stated that the average number of hours of sleep needed by an adult is 8 hours, sleep duration requirements depend on many factors, and there may be a wide spectrum of acceptable sleep physiology in humans. Normative sleep stage data has been published from large data sets [38], but these studies typically focus on what is called summary statistics, such as the percentage of time spent in various sleep-wake stages in the night. This coarse view does not capture much of the rich physiology of sleep. For example, sleep apnea is known to fragment or interrupt REM sleep in many individuals (whether this finding relates to clinical symptoms remains unproven). If one measures the percentage of the night spent in REM sleep in people with severe sleep apnea versus no sleep apnea, there is little or no difference; however, if the time spent in REM sleep is measured through more appropriate methods, called transition analysis, there is clear evidence of fragmentation [39–41]. This concept applies to time spent in any sleep-wake stage, and there is growing evidence that alternative metrics for quantifying sleep-wake stage architecture provide unique insights and may prove more relevant for subjective and medical endpoints than the traditional summary statistics. 

 Despite the attractive idea that certain aspects of sleep are more or less important than others for us to feel refreshed and performing optimally, many challenges remain. For example, the “sleep cycle” length of approximately 90 minutes of alternation between REM and NREM sleep is variable from night to night, and thus that pattern may only be evident upon averaging across multiple nights. The amount of time spent in REM sleep may vary depending on disease states (like sleep apnea), medications (like antidepressants), or alcohol ingestion. Many medications used for sleep have been shown to suppress REM and slow wave sleep and yet may improve the subjective impression of sleep in some individuals. It is important to recognize that much remains unknown in terms of what is normal or optimal, and the answers (if they can be surmised) may even differ from individual to individual. Perhaps the most striking example of individual variability in sleep involves the symptoms of sleep apnea, the best described and most dramatic source of sleep disturbance in the field of sleep medicine. Only half of individuals with severe sleep apnea have daytime sleepiness, whether assessed by subjective report or by objective measurement [42]. The use of home monitoring may allow individuals to attempt to identify patterns of interest that correlate with their own subjective sense. However, caution should be exercised when the output of sleep monitors overlaps with widely held concepts that have little clinical basis (e.g., “The device says I'm not getting enough REM sleep,”) and thus may introduce distractions from self-discovery. 

### 7.6. A Comment on Smart Alarms

 There is clearly a sense of “face validity” for the concept of an alarm clock allowing one to wake up at the optimal time, that is, when sleep is already lightest. Face validity refers to situations in which a concept is so obvious as to obviate the need for validation data. Unfortunately, the history of biomedical research teaches us that most ideas initially felt to have face validity do not withstand the test of rigorous experimentation. Smart alarm claims are not new, and patents based on the idea that one should ideally awaken at a time of light sleep date back over 20 years. The lack of data is concerning, given that this feature is no doubt an important attraction to potential consumers. In fact, how the stage of sleep from which one awakens impacts subjective alertness remains largely unknown. It may be the case that waking from deep, N3 sleep is more difficult and some people may experience sleep inertia when aroused from this stage, and thus alarms that tend to detect periods when one is less likely to be in this stage may be beneficial in terms of avoiding sleep inertia. Testing this would be fairly straightforward. For example, a trial could involve 1-2 weeks of monitoring, in which each morning is randomized to either alarm at the supposedly “optimal” time or at a nonoptimal time. The subject would be blinded to this and would only report their level of alertness upon awakening or how refreshing their sleep was. In this way, one could determine whether the smart-alarm feature was actually serving some benefit at the individual level. Unfortunately, such a trial has not been done, despite multiple devices claiming a smart alarm feature. 

### 7.7. Concluding Comments on a Research Agenda

 The most common measurement technique in the wellness arena is limb movement. Most commercial devices using actigraphy do not have available validation studies. This is a critical limitation given the widespread use of these devices in the wellness arena due to their ease of measurement and simple graphical display of data. It is insufficient to refer, as some devices do, to the rich literature of research-grade wrist actigraphy for two reasons: one is that each device has nuances of movement detection and analysis that cannot be assumed to generalize, and the other is that wrist actigraphy has enjoyed only limited clinical use. For example, actigraphy is mainly used to assess gross sleep-wake patterns over long periods of time in the assessment of circadian rhythm disorders, and in research studies to ensure certain sleep-wake schedules are being adhered to for experimental validity. It is not used to determine sleep stages and is rarely used as a measure of sleep quality outside of research studies. Thus, while the idea that movement-based analysis might prove useful for individuals performing longitudinal tracking is interesting, the limitations of this method should be appreciated. 

The pace of research studies seems to be lagging behind the pace of advertising in the field of home sleep monitors. The M1 device has undergone research in medical contexts and has clearance for use by physicians. Of the products targeting the consumer wellness market, the Zeo headband has published validation data but has not been validated in those with sleep problems (such as insomnia), medical illness, or exposures (medications, alcohol, caffeine), any of which might alter the measured signals and thus confound the built-in analysis. For example, many antidepressant medications are known to alter several aspects of sleep physiology, including muscle tone, eye movements, and EEG rhythms. This is a common problem in general in medical trials: the conclusions may only be relevant for the specific population under the specific conditions of the study. In other words, generalizing the findings of any study to a broader population should be undertaken with caution. It is possible that future developments using analysis of raw data will clarify the strengths and weaknesses of various devices with respect to particular populations. We suggest that the diversity of methods being marketed for sleep-monitoring should be subjected to formal validation studies across a spectrum of populations most likely to benefit, as the algorithms and validity may differ by population. This will be crucial to understand monitoring limitations as well as to maximize the utility of the time and money invested in self-tracking. 
